# Image Encryption Using a New Hybrid Chaotic Map and Spiral Transformation

**DOI:** 10.3390/e25111516

**Published:** 2023-11-05

**Authors:** Mingfang Jiang, Hengfu Yang

**Affiliations:** 1 School of Computer Science, Hunan First Normal University, Changsha 410205, China; bingyuejiang@126.com; 2 Hunan Provincial Key Laboratory of Informationization for Basic Education, Hunan First Normal University, Changsha 410205, China

**Keywords:** image encryption, Chebyshev map, spiral transformation, security analysis

## Abstract

Image encryption based on chaotic maps is an important method for ensuring the secure communication of digital multimedia on the Internet. To improve the encryption performance and security of image encryption systems, a new image encryption algorithm is proposed that employs a compound chaotic map and random cyclic shift. First, a new hybrid chaotic system is designed by coupling logistic, ICMIC, Tent, and Chebyshev (HLITC) maps. Comparison tests with previous chaotic maps in terms of chaotic trajectory, Lyapunov exponent, and approximate entropy illustrate that the new hybrid chaotic map has better chaotic performance. Then, the proposed HLITC chaotic system and spiral transformation are used to develop a new chaotic image encryption scheme using the double permutation strategy. The new HLITC chaotic system is used to generate key sequences used in the image scrambling and diffusion stages. The spiral transformation controlled by the chaotic sequence is used to scramble the pixels of the plaintext image, while the XOR operation based on a chaotic map is used for pixel diffusion. Extensive experiments on statistical analysis, key sensitivity, and key space analysis were conducted. Experimental results show that the proposed encryption scheme has good robustness against brute-force attacks, statistical attacks, and differential attacks and is more effective than many existing chaotic image encryption algorithms.

## 1. Introduction

With the rapid development and wide application of new-generation information technologies, such as cloud computing, big data, the Internet of Things, and artificial intelligence [1,2,3], nowadays, the dissemination of multimedia data, such as digital images, on the Internet has become increasingly prevalent. However, at the same time, rapidly developing digital signal processing technology has brought many security problems to the communication and transmission of digital images on the Internet. Secure transmission and access of digital images in an open network environment have attracted increasing attention from researchers [4,5,6,7]. Image encryption transforms a meaningful image into an unrecognized noise-like image and is an effective method to ensure the security of digital images. Chaos systems have received the attention of researchers because of their inherent properties of sensitivity to initial conditions, ergodicity, and random behavior [8,9].

In recent years, many chaotic image encryption schemes have been proposed. Pareek et al. proposed an image encryption algorithm that employs two logistic maps [10]. In the image encryption scheme, the initial conditions for both logistic maps are derived using an external secret key, and eight different types of operations are exploited to encrypt the image pixels. Given the shortcomings of the small parameter space of the logistic map, Zhang et al. [11] designed an image encryption algorithm by employing discrete exponential chaotic maps to improve image confusion and diffusion. Zhu and Sun [8] presented a modified skew tent map and its application in image encryption. The proposed modified tent map to generate the plaintext-dependent secret key is set. The diffusion operation with cipher output feedback ensures that the cipher image is dependent on both the initial keys and the plaintext image. In [12], the Camellia block cipher and logistic chaotic map are used to encrypt images. It scrambles the image using the cat map and generates the round mask and postwhitening key using the logistic map. However, previous chaos-based image encryption schemes have been built on low-dimensional chaotic maps. Low-dimensional chaotic systems have small key spaces, and the generated chaotic sequence has poor randomness. Kumar et al. employed multiple chaotic maps for image encryption [13]. In this scheme, a logistic map is introduced to shuffle the pixels of the plaintext image, and Arnold’s cat map is used in the diffusion process. Encryption schemes based on low-dimensional chaotic sequences [10,11,12,13] cannot effectively resist statistical attacks.

Hyperchaotic maps have been investigated to design more secure image encryption schemes because of their high randomness and ergodicity. Ref. [14] proposed a Lorenz-based image encryption algorithm with a perceptron model. It extended the periodicity of the Lorenz chaotic map by dynamically adjusting the parameters of the chaotic system. The modified Lorenz chaotic map is used to produce three sets of pseudorandom sequences. Akhshani et al. presented a hierarchy of 2D piecewise chaotic maps with an invariant measure and developed a new image encryption scheme using the significant properties of these chaotic maps, such as ergodicity, sensitivity to the initial condition, and random-like behavior [15]. The famous Chen hyperchaotic system was used to generate the shuffling matrix and the diffusing matrix in Ref. [16]. First, the Chen hyperchaotic system is used to shuffle the position of the image pixels. Hua et al. built a new 2D chaotic system (called 2D-LSM) using logistic and sine maps, and the 2D-LSM chaotic map was further used to devise a new image encryption algorithm based on the image content [17]. To enhance encryption effectiveness, Ye et al. proposed a chaotic encryption scheme that combines a 3D logistic map and the secure hash algorithm-3 (SHA-3) [18]. Wang et al. proposed a color image encryption method that employs 4D chaotic maps and DNA encoding [19]. In the encryption scheme, the rules of DNA encoding are controlled by four chaotic sequences generated by the new 4D chaotic system. Gong et al. designed a new 4D chaotic system with coexisting asymmetric attractors [20]. Theoretical analysis of the phase portrait, bifurcation diagram, and Lyapunov exponent and its applications in random number generator (RNG) and image encryption verified the feasibility of the new 4D chaotic map.

Recently, compound chaotic systems have been an effective approach for image encryption [21,22,23] because they show better randomness and noise-like behaviors than other chaotic systems. Zhu et al. proposed a 2D composite discrete chaotic system (CDCS) [24]. To obtain a good permutation effect, the new CDCS system is used for bit-level permutation and pixel-level diffusion. In 2017, they developed another compound homogeneous hyperchaotic system (CHHCS) that is employed to permutate plaintext images [25]. In addition, dynamic LBP operations are used to diffuse each permutated pixel. Hua et al. introduced a cosine-transform-based chaotic system (CTBCS) [26]. CTBCS is exploited to generate three chaotic maps. One of these maps is used to design an image encryption algorithm where high-efficiency scrambling is used to separate adjacent pixels and random order substitution is used to spread a small change in the plaintext image to all pixels of the cipher image. Gao et al. presented an image encryption algorithm by coupling the sine and tent maps [27]. Bit rearrangement is used to further improve the composite sine–tent map. Image pixels are encrypted by applying the most significant bit substitution, scrambling, and diffusion. To overcome bandwidth and security issues simultaneously, Yadav et al. devised a joint image compression and encryption scheme using hybrid chaotic maps [28]. The absolute moment transcribed coding approach is used for compression, and Arnold’s cat and Henon maps are applied to the compressed image for encryption. To improve the chaotic characteristics, Zhang and Liu [29] designed a compound sine–piecewise linear chaotic map (SPWLCM) and proposed an image encryption algorithm using SPWLCM chaos and DNA coding. Wang and Du developed two chaotic systems: 1D logistic Chebyshev map (1DLCM) and logistic Chebyshev coupled map lattices (LCDCML) [30]. LCDCML uses 1DLCM as the dynamic coupling coefficient and further proposes a pixel-level and bit-level image encryption algorithm using these two new chaotic systems. In the encryption scheme, image scrambling and diffusion processes are implemented with chaotic sequences generated by the LCDCML map. Basha et al. presented a bit-level color image encryption scheme using a logistic–sine–tent–Chebyshev (LSTC) map [31]. The LSTC map, cyclic shifts, and XOR operation are used for the mutual diffusion of the two color components. The binary element is exchanged and transformed into another binary bit plane using the LSTC map.

Two main issues are discussed in this paper: (1) To obtain better randomness and ergodicity, we construct a new hybrid chaotic map by coupling multiple chaotic maps and (2) design a new image encryption application to enhance the security of image encryption. The main contributions of this paper are summarized as follows: (1) We developed a new hybrid chaotic map by coupling logistic, ICMIC, tent, and Chebyshev maps (called HLITC). Performance evaluations by chaotic trajectory, Lyapunov exponent, and Kolmogorov entropy testify that the new hybrid chaotic map has better key sensitivity to the initial value and larger control parameter space. (2) A chaotic image encryption algorithm using the presented HLITC system is proposed. During encryption, key sequences generated by the HLITC map are used for image scrambling and diffusion stages. The spiral transformation controlled by the HLITC map is employed to scramble the pixels of the plaintext image, and the XOR operation dependent on the chaotic map is used for image diffusion. (3) The experiments demonstrate that the proposed encryption algorithm has high resistance to statistical differential and brute-force attacks, and it can achieve higher security than several previous chaotic image encryption algorithms.

The rest of the paper is organized as follows. Section 2 introduces classic chaotic maps and the new hybrid chaotic map. The new image encryption algorithm based on the HLITC system is presented in Section 3. We discuss the experimental results in Section 4 and conclude in Section 5.

## 2. Construction of the Hybrid Chaotic Map

This section presents the new hybrid chaotic system by coupling logistic, ICMIC, tent, and Chebyshev maps (HLITC) and their properties. To prove the superiority of the HLITC system, a comparison between three sample chaotic maps is conducted in terms of chaotic trajectory, Lyapunov exponent, and Kolmogorov entropy.

### 2.1. Classic Chaotic Maps

#### 2.1.1. Logistic Map

**Figure 1:**
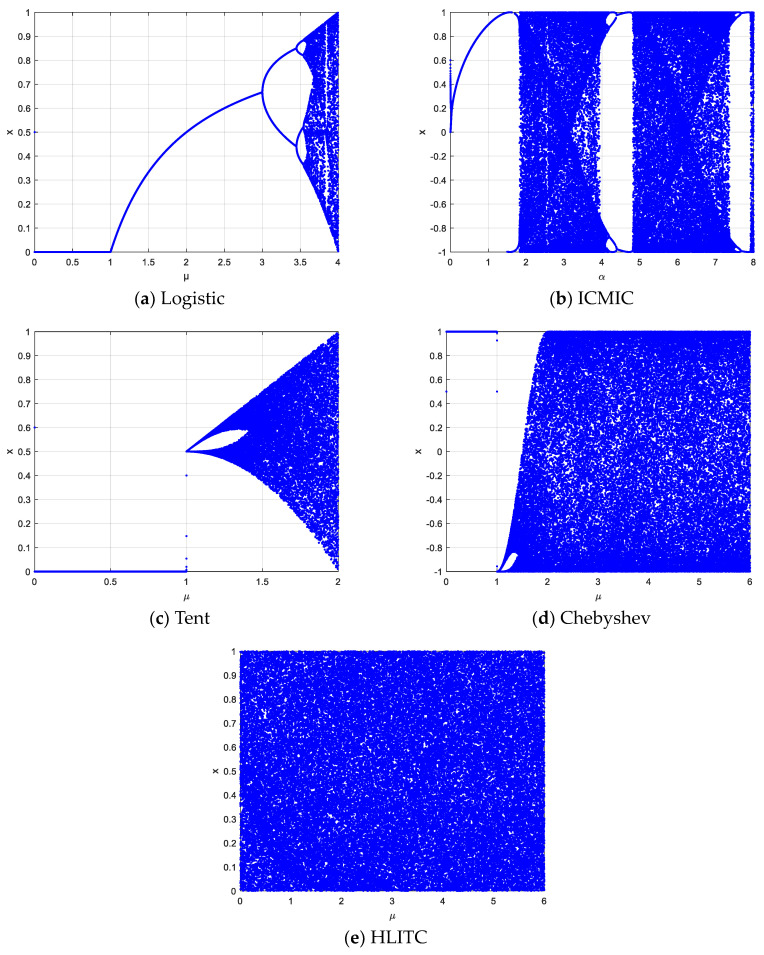
Dynamics comparison with bifurcation diagrams: (**a**) logistic, (**b**) ICMIC, (**c**) tent, (**d**) Chebyshev, and (**e**) HLITC.

The logistic map is a quadratic polynomial map, which is a typical map representing complex nonlinear behavior. The mathematical expression is written as follows:

(1) $$x_{n+1}=\mu x_{n}\left(1-x_{n}\right),\mu \in \left[0,4\right],x_{n}\in \left(0,1\right)$$

where *μ* is the bifurcation parameter. Only when 3.5699456 < *μ* ≤ 4, the logistic map falls into a chaotic state. The bifurcation diagram of the logistic map is shown in Figure 1a.

#### 2.1.2. ICMIC Map

He et al. proposed a 1D iterative chaotic map with infinite collapses (ICMIC) [32]. Compared with the logistic and tent maps, the ICMIC map has the advantages of uniform traversal and fast convergence. It can be expressed as

(2) $$x_{n+1}=sin\left(\alpha /x_{n}\right),\alpha \in \left[0,\infty \right],x_{n}\in \left[-1,0),]\cup (0,1\right]$$

Figure 1b shows the corresponding bifurcation diagram.

#### 2.1.3. Tent Map

Tent maps are piecewise linear maps with a relatively uniform distribution. The bifurcation diagram for the tent map is shown in Figure 1c. It has been widely used in chaotic cryptography. Its mathematical expression is as follows:

(3) $$x_{n+1}=\left\{\begin{array}{l} \mu x_{n},\;\;\;\;x_{n}\in \left(0,0.5\right)\\ \mu \left(1-x_{n}\right),\;\;x_{n}\in [0.5,1) \end{array}\right.$$

where μ is the control parameter, and $\mu \in \left(0,2\right)$.

#### 2.1.4. Chebyshev Map

The Chebyshev map is one of the 1D chaotic maps with good nonlinear dynamic characteristics. Figure 1d shows the bifurcation diagram for the Chebyshev map. When the control parameter μ is greater than 1, chaos occurs. When greater than 2, the map is in a chaotic state. It can be defined as follows:

(4) $$x_{n+1}=cos\left(\mu \cdot arccosx_{n}\right),x_{n}\in \left[-1,1\right]$$

### 2.2. Proposed HLITC Chaotic System

To enhance the randomness of chaotic systems, we designed a new chaotic system by combining the logistic map, ICMIC map, tent map, and Chebyshev map. The main process of building the chaotic system is as follows:

First, with the compound ICMIC map and logistic map, we have

(5) $$x_{n+1}=sin\left(\frac{\mu \pi }{\mu x_{n}\left(1-x_{n}\right)}\right)$$

To skip blank windows further, we change the parameter μ to $\left(\frac{\mu }{4}+3.6\right)$. The compound ICMIC map can be rewritten as

(6) $$x_{n+1}=sin\left(\frac{\left(\frac{\mu }{4}+3.6\right)\pi }{\mu x_{n}\left(1-x_{n}\right)}\right)$$

Then, the Chebyshev map and the tent map are compounded, and the control parameter μ by μ+2 is modified to avoid falling into blank areas. The compounded Chebyshev chaotic map is expressed as follows:

(7) $$x_{n+1}=\left\{\begin{array}{l} cos\left(\left(\mu +2\right)arccos\left(4\mu x_{n}\right)\right),\;\;\;\;x_{n}\in \left(0,0.5\right)\\ cos\left(\left(\mu +2\right)arccos\left(4\mu \left(1-x_{n}\right)\right)\right),\;\;x_{n}\in [0.5,1) \end{array}\right.$$

Finally, to improve the randomness of the chaotic system, a modular function is used to integrate the compound Chebyshev map and the improved ICMIC map, and a hybrid HLITC chaotic system is produced. Its mathematical definition is as follows:

(8) $$x_{n+1}=\left\{\begin{array}{l} mod\left(sin\left(\frac{\left(\frac{\mu }{4}+3.6\right)}{\mu x_{n}\left(1-x_{n}\right)}\right)+cos\left(\left(\mu +2\right)arccos\left(\left(\mu +3.6\right)x_{n}\right)\right),1\right),\;\;\;\;x_{n}\in \left(0,0.5\right)\\ mod\left(sin\left(\frac{\left(\frac{\mu }{4}+3.6\right)}{x_{n}}\right)+cos\left(\left(\mu +2\right)arccos\left(\left(\mu +3.6\right)\left(1-x_{n}\right)\right)\right),1\right),\;\;x_{n}\in [0.5,1) \end{array}\right.$$

The improved HLITC map makes the chaotic sequence distribution more uniform and has a large parameter space range from μ∈[0,∞). It can avoid the stability window and the blank area. The bifurcation diagram is illustrated in Figure 1e. From Figure 1, it can be seen that the HLITC map has a larger parameter space and better randomness than traditional chaotic maps.

### 2.3. Lyapunov Exponent

The Lyapunov exponent is an important indicator of the dynamic behavior of nonlinear systems. In this section, the Lyapunov exponent (LE) is further used to analyze the predictability of the HLITC chaotic system. This can be written as

(9) $$\lambda =\underset{n\to \infty }{\lim}\left[\frac{1}{n}\sum_{n=0}^{N-1}\ln\left|\frac{{dx}_{n+1}}{{dx}_{n}}\right|\right]$$

where $x_{n}$ is the *n*th iteration value, and *N* is a suitably large integer.

**Figure 2:**
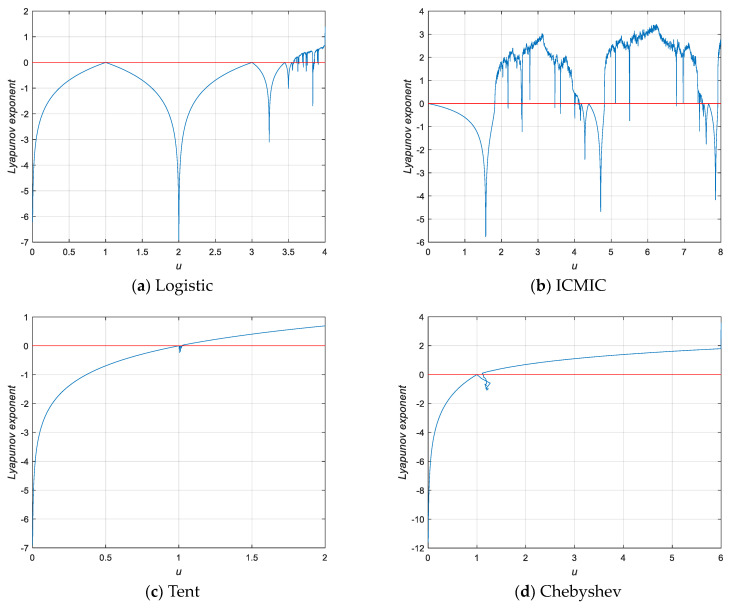

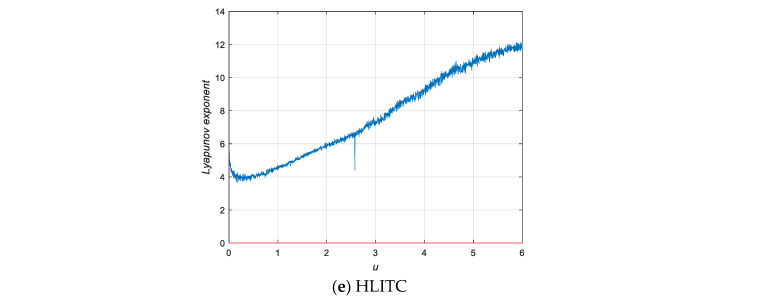
Lyapunov exponent diagrams of different chaotic maps: (**a**) logistic, (**b**) ICMIC, (**c**) tent, (**d**) Chebyshev, and (**e**) HLITC.

Figure 2 shows a comparison between logistic, ICMIC, tent, Chebyshev, and HLITC for the parameter Lyapunov exponent. From Figure 2, it can be seen that all LE values of the HLTIC map for all control parameter values are greater than 0. Therefore, the HLITC map has more chaotic behaviors than the classic chaotic maps in terms of LE.

### 2.4. Information Entropy

Information entropy is usually used to measure the uncertainty of a variable. The greater the uncertainty of variables, the higher the information entropy, which is defined as

(10) $$H\left(X\right)=\sum_{i=0}^{L-1}p\left(x_{i}\right)log\frac{1}{p\left(x_{i}\right)}$$

where $X=\left\{x_{i}|x_{i}\in \left[0,255\right]\right\}$, $p\left(x_{i}\right)$ is the probability of $x_{i}$, and *L* = 256 for grayscale images. The maximum value of information entropy is 8 for grayscale images.

**Figure 3:**
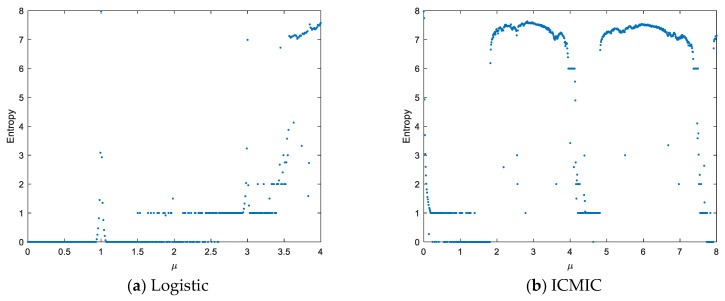

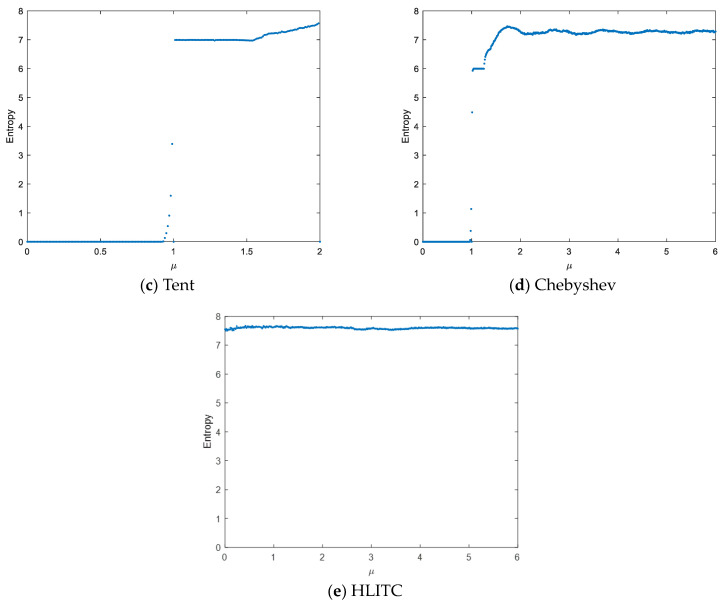
Dynamics comparison of different chaotic maps in terms of information entropy.

Figure 3 shows the information entropy diagrams of the different chaotic maps. It can be seen that the entropy values of output sequences generated by the proposed HLITC map are close to the ideal value 8, which indicates that the HTLITC map has better unpredictability than the abovementioned traditional chaotic maps.

### 2.5. Approximate Entropy

Approximate entropy (ApEn) is a statistical metric that quantifies the regularity and complexity of time series data. The more complex the time series, the greater the corresponding approximate entropy of the time sequence.

For the chaotic sequence $S=\left\{s\left(1\right),s\left(2\right),\ldots ,s\left(N\right)\right\}$ containing *N* data points, the approximate entropy is calculated as follows:

(11) $$ApEn\left(m,r,N\right)=\phi_{m}\left(r\right)-\phi_{m+1}\left(r\right)$$

where

(12) $$\phi_{m}\left(r\right)=\frac{1}{N-m+1}\sum_{i=1}^{N-m+1}\frac{\left|\left\{j\left|d\left(x\left(i\right),x\left(j\right)\right)<r\right.\right\}\right|}{N-m+1}$$

**Figure 4:**
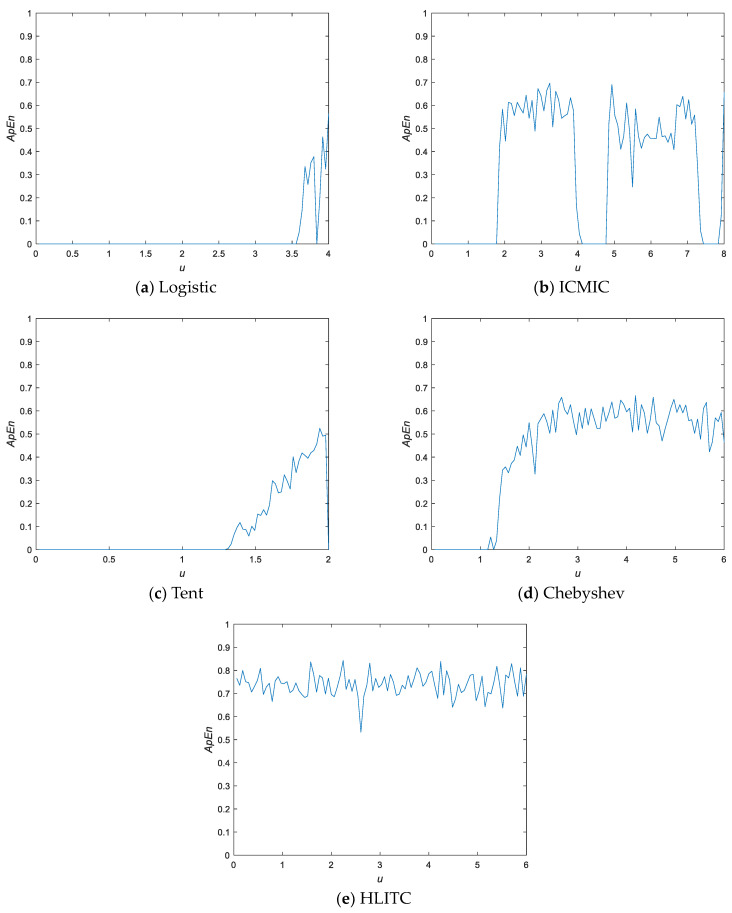
Approximate entropy curves.

Figure 4 shows the approximate entropy curves of five different chaotic maps. As shown in Figure 4, the complexities of the four previous chaotic maps fluctuate greatly. For example, all the other four previous chaotic maps have an ApEn value of 0 when the parameter μ<1. However, for the proposed HLITC chaotic map, the ApEn values are greater than 0.5 for the parameters μ>0. Therefore, the HLITC map has good chaotic properties.

### 2.6. Randomness Testing

To further test the randomness of the HLITC map, the NIST SP800 test was performed on the chaotic sequences generated by the HTLITC system. The NIST randomness testing has 15 performance tests that are accessed by *p*-value. If the *p*-value is less than 0.01, the randomness of the tested sequence is poor, and if the *p*-value is greater than or equal to 0.01, the randomness of the tested sequence is good.

In the testing, the chaotic sequence generated by the HLITC map is first converted into a binary sequence, and then NIST randomness testing is performed. The test results are listed in Table 1. From Table 1, it can be seen that the chaotic sequence produced by the new HLITC system has good randomness among all 15 indicators tested by NIST, and all *p*-values are greater than 0.01, indicating that the generated chaotic sequence has good randomness.

## 3. Image Encryption Based on a Hybrid HLITC Map

**Figure 5:**
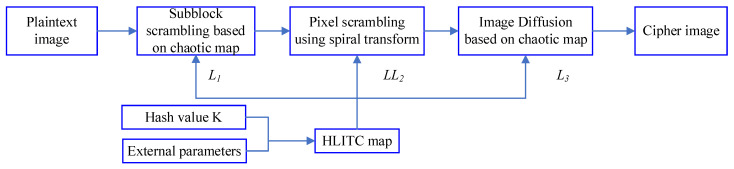
Image encryption process.

This section introduces the proposed image encryption algorithms based on spiral transformation and chaos maps. The proposed image encryption algorithm based on the HLITC map is composed of three stages: key generation, image scrambling, and image diffusion, as illustrated in Figure 5.

### 3.1. Key Generation

To enhance security and enlarge the key space, the hash value of the plaintext image and external parameters are employed to produce the control parameters and initial values of the proposed HLITC map. Assume that the original image is denoted as $I_{m\times n}$. The detailed process of key generation is illustrated as follows.

Step 1: Obtain the hash value *K* of the plaintext image by applying the SHA-256 hash function.

Step 2: Divide the hash value *K* into 32 segments with each of one byte. Thus, *K* can be expressed as $K=k_{1}$,$k_{2},\ldots ,k_{i},\ldots ,k_{32}$, where *I* = 1, 2, …, …, 32, and $k_{i}$ is an integer in the interval [0, 255].

Step 3: The initial keys $\lambda_{i}$, *I* = 1, 2, 3 are used as the external parameters, and then the initial values $x_{i}$ and control parameters $\mu_{i}$ are generated.

(13) $$\left\{\begin{array}{l} x_{i}=\frac{mod\left(k_{6\left(i-1\right)+1}⊗k_{6\left(i-1\right)+2}⊗k_{6\left(i-1\right)+3}⊗k_{6\left(i-1\right)+4}⊗k_{6\left(i-1\right)+5}⊗k_{6i}⊗k_{31}+\lambda_{i},256\right)}{256}\\ \mu_{i}=mod\left(\sum_{j=1}^{5}k_{6\left(i+2\right)+j}+k_{32}+\lambda_{i},256\right) \end{array}\right.$$

where ⊗ denotes exclusive OR operation.

Step 4: Iterate the HLITC map for $n_{0}+num$ times using $x_{i}$ and $\mu_{i}$ as the initial values and control parameters and generate three chaotic sequences $y_{1}$, $y_{2}$, $y_{3}$. Their length num is $N_{b}$, $N_{b}$*,* and m×n, respectively. To minimize the aperiodic effect, the first $n_{0}$ values are discarded, where m×n is the size of the original image.

Step 5: Produce three random integer sequences $L_{1},L_{2},{andL}_{3}$ for image scrambling and image diffusion.

(14) $$L_{1}=mod\left(round\left(y_{1}\times 10^{15}\right),N_{b}\right)+1$$

(15) $$L_{2}=mod\left(round\left(y_{2}\times 10^{15}\right),B\right)+1$$

(16) $$L_{3}=mod\left(round\left(y_{3}\times 10^{15}\right),256\right)$$

where *B* is the number of elements of an image subblock, and $N_{b}$ is the number of image subblocks.

(17) $$N_{b}=\frac{m\times n}{B}$$

### 3.2. Image Encryption

In this method, to enhance the pseudorandomness of image scrambling, arbitrary point spiral transformation based on a chaotic map is used for image scrambling, and the XOR operation of chaotic control is introduced in the pixel diffusion process to further improve the encryption effect.

#### 3.2.1. Image Scrambling Based on Spiral Transformation

The image scrambling process consists of image subblock scrambling based on chaos control and pixel scrambling based on the spiral transformation of arbitrary points. The detailed scrambling process is as follows:

Step 1: Divide the plaintext image $I_{m\times n}$ into subblock of size s×s, i.e., B=s×s. Denote the sequence of all subblock as *Q*.

Step 2: Use the chaotic sequence $L_{1}$ of length $N_{b}$ to scramble the image subblock ${Ib}^{i}$, and the scrambled subblock sequence $Q^{\prime }$ is obtained.

(18) $$Q^{\prime }=Q\left(L_{1}\right)$$

Step 3: Generate the chaotic sequence ${LL}_{2}$ (as shown in Equation (19)) by using the sequence $L_{2}$ and the average of each image subblock ${Ib}^{i}$ in $Q^{\prime }$.

(19) $${LL}_{2}=mod\left(round\left(y_{2}\times \frac{ave}{255}\times 10^{15}\right),B\right)+1$$

where *ave* is the average of each image subblock ${Ib}^{i}$.

Step 4: According to the random starting point $L_{2}\left(i\right)$, we perform the spiral transformation on all pixels within each image subblock to obtain the scrambled image $I^{\prime }$.

**Figure 6:**
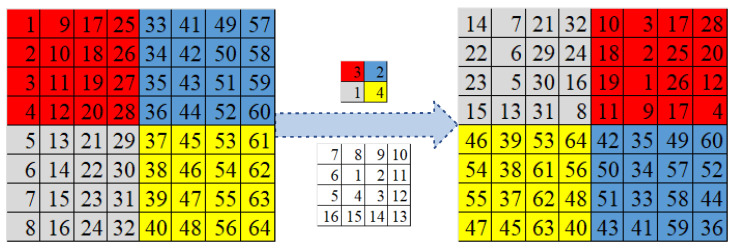
Example of image scrambling based on a spiral transformation: s = 4, $N_{b}=4$.

Figure 6 shows an example of image scrambling based on spiral transformation.

#### 3.2.2. Image Diffusion Based on a Chaotic Map

Step 1: Read scrambled image data *P* from scrambled image $I^{\prime }$.

Step 2: Reshape matrix *P* to sequence $P^{\prime }$ of length m×n.

Step 3: Perform the R-round XOR operation for image diffusion. The detailed process of each round of XOR operation is written as follows.

(20) $$E\left(i\right)=\left\{\begin{array}{l} P^{\prime }\left(i\right)⨂L_{3}\left(i\right)⨂E\left(1\right),i=1\\ P^{\prime }\left(i\right)⨂L_{3}\left(i\right)⨂E\left(i-1\right),i>1 \end{array}\right.$$

where $E\left(1\right)=mod\left(round\left(\frac{\sum_{i=1}^{32}k_{i}}{32}\times 10^{15}\right),256\right)$.

Step 4: Reshape sequence *E* into a matrix of size m×n to obtain the final ciphertext image $I_{e}$.

The proposed image encryption system is symmetric, and the decryption algorithm is the inverse of the encryption algorithm.

## 4. Experimental Results

**Figure 7:**
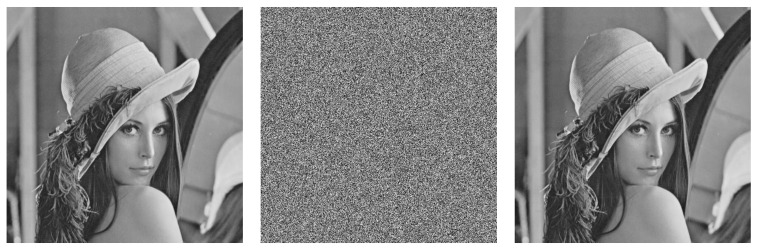

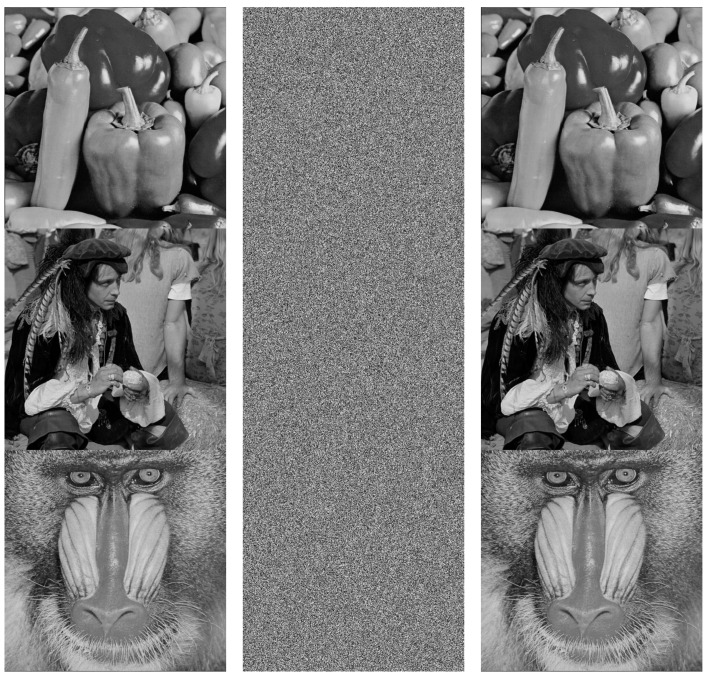
Examples of image encryption and decryption. (**left** column): plaintext image, (**center** column): cipher image, and (**right** column): decipher image (top to down: Lena, peppers, man, and baboon).

In the experiment, some grayscale images of size 512 × 512 were used to evaluate encryption performance. The simulated experiments were performed on an Intel Core I i7-12700H CPU (Intel Corporation, California, USA) with 16 G memory. The version of MATLAB used was R2020a. Figure 7 illustrates the results of image encryption and decryption using the proposed encryption algorithm. Figure 7 shows that the ciphertext image is an irregular noise signal image, and it is difficult to observe the relevant information from the ciphertext image. However, the decrypted image is consistent with the plaintext image, indicating that the proposed encryption method has good encryption performance.

### 4.1. Histogram Analysis

**Figure 8:**
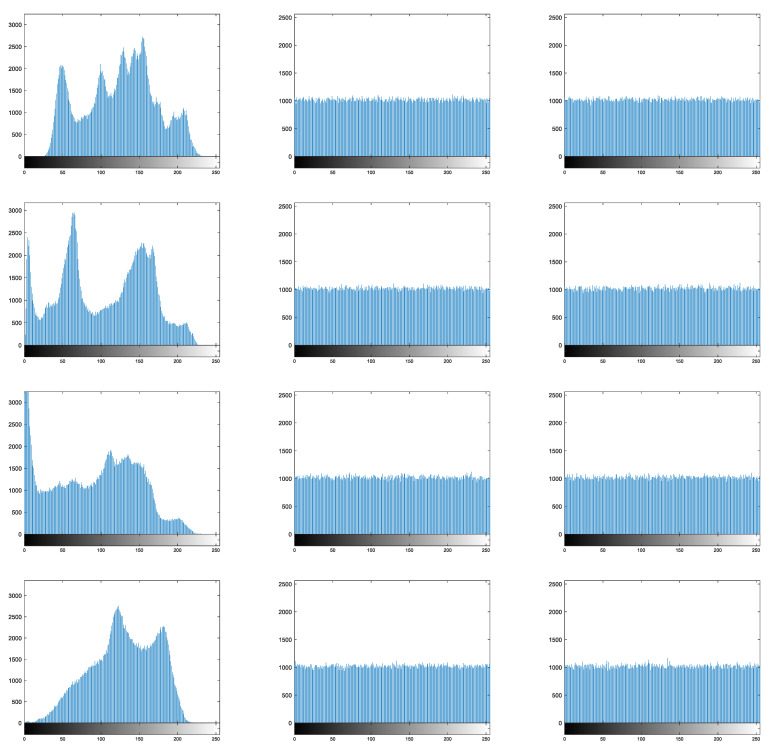
Histogram analysis (top to down: Lena, peppers, man, and baboon).

The image histogram is the probability statistics of the image pixels, and the ideal distribution of the ciphertext image pixels should be uniform. Figure 8 shows a comparison of image histograms before (right column) and after encryption (center column). Histograms of the existing encryption algorithms [33] are shown in the right column. Both our encryption scheme and Ref. [33] have flat histograms of the ciphertext images. However, for the baboon image, the histogram of the ciphertext images obtained from our method is relatively flatter.

Figure 8 shows that the histogram distribution of plaintext images is uneven, whereas the histogram of ciphertext images is uniformly distributed, indicating that the proposed method has good resistance to statistical attacks. In addition, the histogram variance is used to quantitatively measure the uniformity of the pixel distribution in ciphertext images. The smaller the value of the histogram variance, the more uniform the distribution of ciphertext images and the higher the security of the encryption method.

To test the effectiveness of our encryption method, three previous chaos-based image encryption schemes [30,33,34] were implemented.

Ref. [30]: In this paper, two new chaotic systems were proposed. One was a one-dimensional logistic Chebyshev map (1DLCM) and the other was a logistic Chebyshev dynamic coupled map lattice (LCDCML).

Ref. [33]: An efficient image encryption approach based on simultaneous permutation and diffusion functions was proposed. It employs the Chebyshev map to scramble the plaintext images and the modified logistic map to diffuse the image pixels.

Ref. [34]: A new chaos image encryption algorithm was proposed using the Hilbert curve and the Henon map. Pixel-level and bit-level permutations based on the cyclic shift operation are employed for image confusion.

The histogram variance test results of the different encryption methods for ciphertext images are shown in Table 2. From Table 2, it can be seen that the histogram variance of the ciphertext image obtained by our encryption method is smaller than that of the existing encryption methods, indicating that it has good security.

### 4.2. Correlation Analysis

**Figure 9:**
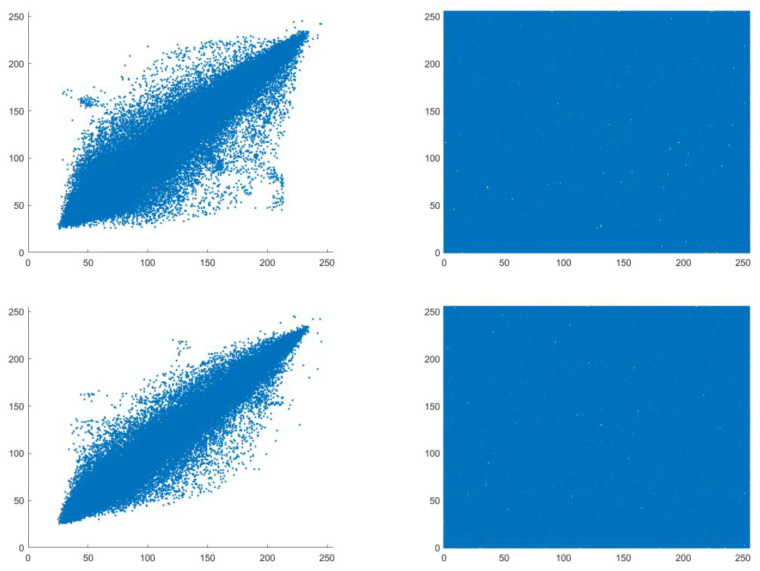

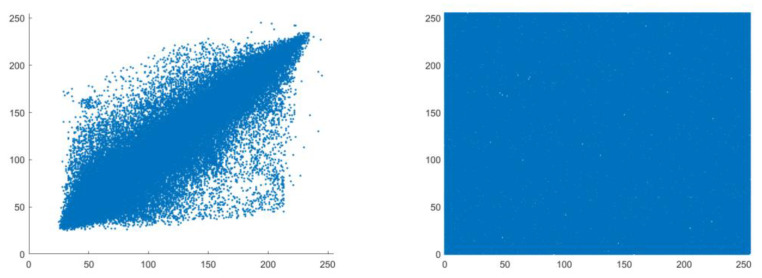
Pixel correlation between the Lena image and its ciphertext image in various directions ((**Left**): plaintext image (**Right**): ciphertext image, top to down: horizontal, vertical, and diagonal).

Rich relationships and dependencies exist between adjacent pixels in original images, resulting in high correlation. Therefore, eliminating the correlation between adjacent pixels is one of the key requirements for ensuring the security of image encryption methods. Cipher images with low correlation in the horizontal, vertical, and diagonal directions are considered effective in resisting statistical attacks. The visual results of the correlation before and after encryption are shown in Figure 9. Figure 9 shows that the scatter points related to adjacent pixels in the plaintext image are relatively concentrated, while the ciphertext image is uniformly distributed, indicating that the encryption algorithm effectively eliminates the correlation in all directions of the plaintext images.

### 4.3. Analysis of Differential Attack

A differential attack means that an attacker finds the corresponding relationship between the plaintext image and the ciphertext image by comparing the differences between the corresponding ciphertext before and after slightly changing the plaintext. The ability of algorithms to resist differential attacks is generally evaluated using two indicators: number of pixels change rate (NPCR) and unified average change intensity (UACI).

(21) $$C\left(i,j\right)=\left\{\begin{array}{l} 0,\;ifI_{1}\left(i,j\right)=I_{2}\left(i,j\right)\\ 1,\;else \end{array}\right.$$

(22) $$NPCR=\frac{\sum_{i=1}^{m}\sum_{j=1}^{n}C\left(i,j\right)}{m\times n}\times 100\%$$

(23) $$UACI=\frac{\sum_{i=1}^{m}\sum_{j=1}^{n}\left|I_{1}\left(i,j\right)-I_{2}\left(i,j\right)\right|}{m\times n\times 255}$$

For A pixel from the Lena plaintext image was randomly selected, and its value was increased by 1. Using the proposed encryption method, the encryption experiments were performed 1000 times, and different pixels were chosen each time. The average NPCR and UACI values were calculated, and the testing results are shown in Table 3.

From Table 3, it can be seen that the NPCR and UACI values of the ciphertext images using our algorithm all exceed 99.66 and 33.58, respectively. Compared with other chaotic image encryption algorithms, our algorithm has slightly higher average NPCR and UACI values, indicating that it can more effectively resist differential attacks.

### 4.4. Information Entropy Analysis

Information entropy reflects the uncertainty of image information. In general, the larger the entropy, the greater the amount of information, and the less detectable information. For 256-level grayscale images, the theoretical value of information entropy is 8. Table 4 records the information entropy before and after image encryption and compares it with other algorithms. The results show that the ciphertext image generated by the proposed algorithm can effectively conceal information.

### 4.5. Running Time

To evaluate the effectiveness of the proposed image encryption scheme, the time cost of the algorithm was tested. In the test, the 512 × 512 grayscale images were used for encryption and decryption. The average encryption and decryption times of 10 experiments were calculated, and the results are listed in Table 5.

The test results demonstrate that the proposed encryption scheme is a cost-effective image encryption scheme that can be used for image encryption in cloud computing.

## 5. Conclusions

This paper designs a composite Chebyshev chaotic map (HLITC), and random testing shows that the composite HLITC map has good randomness. Subsequently, a new image encryption algorithm is proposed by combining the HLITC map with spiral transformation. First, the initial values of the compound HLITC chaotic system are generated using the SHA-256 hash function and plaintext images and are used for generating chaotic sequences. Then, a random starting point spiral transformation is introduced to eliminate the periodicity of chaotic systems. Spiral transformation is used for scrambling, and image diffusion is achieved by combining chaos and XOR operation. The experimental results show that the proposed algorithm can effectively hide plaintext information, has strong key sensitivity, and can resist differential attacks and other attack methods. Moreover, it has both low encryption and low decryption costs and is suitable for secure communication of image data in the cloud computing environment.

## Figures and Tables

**Table 1:** Results of the NIST randomness test for HLITC.

Test Name	*p*-Value	Result
Approximate entropy test	0.9548	Success
Block frequency test	0.0383	Success
Cumulative sums (forward) test	0.2631	Success
FFT test	0.2789	Success
Frequency test	0.2919	Success
Linear complexity test	0.8934	Success
Longest runs of one test	0.4398	Success
Nonoverlapping template matching test	0.9961	Success
Overlapping template matching test	0.4771	Success
Binary matrix rank test	0.9562	Success
Runs test	0.3959	Success
Serial test	0.0297	Success
Maurer’s universal statistical test	0.4439	Success
Random excursion test	0.3061	Success
Random excursion variant test	0.0527	Success

**Table 2:** Histogram variance analysis of the ciphertext image.

Encryption Method (Ciphertext Image)	Histogram Variance
[33] (Lena)	242.4651
[34] (Lena)	124.6218
[30] (Lena)	68.9023
Our method (Lena)	31.5731
Our method (peppers)	29.7752
Our method (man)	32.6184
Our method (baboon)	30.2543

**Table 3:** Comparison of the NPCR and UACI results.

Metric	Our Method	Ref. [33]	Ref. [34]
NPCR	99.6645	99.6052	99.6226
UACI	33.58	33.28	33.14

**Table 4:** Information entropy comparison of the ciphertext images.

Plaintext Image	Our Scheme	Ref. [33]	Ref. [34]
7.4455	7.9986	7.9935	7.9926

**Table 5:** Comparison of encryption and decryption times for different images (second).

Stage	Lena	Peppers	Man	Baboon
encryption	0.2565	0.2779	0.2761	0.2760
decryption	0.1566	0.1674	0.1589	0.1498

## Data Availability

Not applicable.
